# 2-Phenyl-1,3-selenazole-4-carb­oxy­lic acid

**DOI:** 10.1107/S1600536811007185

**Published:** 2011-03-05

**Authors:** Jin-Bei Shen, Xin Lv, Ji-Fei Chen, Yu-Feng Zhou, Guo-Liang Zhao

**Affiliations:** a College of Chemistry and Life Science, Zhejiang Normal University, Jinhua 321004, Zhejiang, People’s Republic of China; b Zhejiang Normal University Xingzhi College, Jinhua, Zhejiang 321004, People’s Republic of China

## Abstract

In the title compound, C_10_H_7_NO_2_Se, the two rings are twisted, making a dihedral angle of 12.42 (9)°. In the crystal, pairs of mol­ecules are disposed about an inversion center, generating O—H⋯O hydrogen-bonded dimers.

## Related literature

For the synthesis, see: Zhao *et al.* (2010 ▶). For related structures, see: Srivastava & Robins (1983 ▶); Boritzki *et al.* (1985 ▶); Shen *et al.* (2011 ▶).
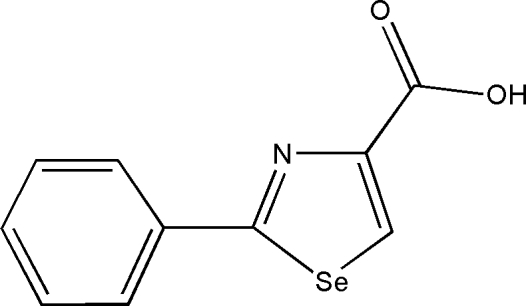


## Experimental

### 

#### Crystal data

C_10_H_7_NO_2_Se
*M* *_r_* = 252.13
Monoclinic, 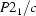

*a* = 8.0817 (3) Å
*b* = 11.5661 (4) Å
*c* = 11.6295 (4) Å
β = 117.168 (2)°
*V* = 967.12 (6) Å^3^
*Z* = 4
Mo *K*α radiation
μ = 3.85 mm^−1^
*T* = 296 K
0.23 × 0.22 × 0.19 mm

#### Data collection

Bruker APEXII area-detector diffractometer
Absorption correction: multi-scan (*SADABS*; Sheldrick, 1996 ▶) *T*_min_ = 0.437, *T*_max_ = 0.479
7502 measured reflections
1705 independent reflections
1487 reflections with *I* > 2σ(*I*)
*R*_int_ = 0.022

#### Refinement

*R*[*F* ^2^ > 2σ(*F* ^2^)] = 0.024
*wR*(*F* ^2^) = 0.063
*S* = 1.05
1705 reflections
127 parameters
H-atom parameters constrained
Δρ_max_ = 0.43 e Å^−3^
Δρ_min_ = −0.19 e Å^−3^

### 

Data collection: *APEX2* (Bruker, 2006 ▶); cell refinement: *SAINT* (Bruker, 2006 ▶); data reduction: *SAINT*; program(s) used to solve structure: *SHELXS97* (Sheldrick, 2008 ▶); program(s) used to refine structure: *SHELXL97* (Sheldrick, 2008 ▶); molecular graphics: *SHELXTL* (Sheldrick, 2008 ▶); software used to prepare material for publication: *SHELXL97*.

## Supplementary Material

Crystal structure: contains datablocks I, global. DOI: 10.1107/S1600536811007185/ng5124sup1.cif

Structure factors: contains datablocks I. DOI: 10.1107/S1600536811007185/ng5124Isup2.hkl

Additional supplementary materials:  crystallographic information; 3D view; checkCIF report

## Figures and Tables

**Table 1:** Hydrogen-bond geometry (Å, °)

*D*—H⋯*A*	*D*—H	H⋯*A*	*D*⋯*A*	*D*—H⋯*A*
O1—H7⋯O2^i^	0.82	1.81	2.623 (2)	171

Symmetry code: (i) 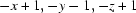.

**Fig. 1.**
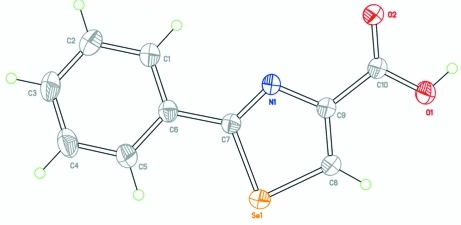
The molecular structure of the title complex, showing the atom- labeling scheme. Displacement ellipsoids are drawn at the 30% probability level.

**Fig. 2.**
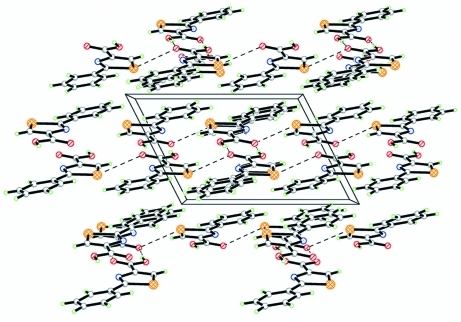
The stacking plot of the title compound, showing H-bond interactions (dashed lines) and π···π stacking interactions.

## supplementary crystallographic information

### Comment

It has well been confirmed that the derivatives of selenazole are important in multiple fields such as chemistry and biochemistry owing to their biological activities (Srivastava *et al.*, 1983;Boritzki *et al.*,1985). Interested in this field, we have been engaged in a major effort directed toward the development of syntheses of new selenazole carboxylic acid and their transition metal complexes. In a few of articles we have reported our partial research results (Zhao *et al.*, 2010;Shen *et al.*, 2011). Herein,we crystallize the organic ligand 2-phenyl-4-selenazole carboxylic acid.

The structure of the title, (C_10_H_7_NO_2_Se),suitable for X-ray, was obtained by chance. The structure of the complex is shown in Fig.1, which reveals that all atoms in each molecule are nearly coplanar in the centrosymmetric unit. The molecule is essentially planar with the dihedral angle between two neighboring rings are 12.415 (89)°. In the molecule of 2-phenyl-4-selenazole carboxylic acid,the Se—C bond length range from 1.832 (2) Å-1.891 (2)Å and the angle C—Se—C is 84.78 (10)°.

The molecules arranged in the crystal at regular intervals with O—H···O hydrogen bonds. The end to end hydrogen-bonding interactions lead to the formation a one-dimensional structure framework along the *b* axis, Fig 2. Between adjacent triple-helix chains there exist weak π···π interactions.

### Experimental

Reagents and solvents used were of commercially available quality and without purified before using. K_2_Cr_2_O_7_ (0.588 g, 2 mmol) was added to a mixed solution of acetic acid (50 ml) with 2-phenyl-4-selenazole carbinol (0.248 g, 1 mmol) under stirred conditions at room temperature. Few minutes later lots of red deposit appeared. After the deposit was filtered out, a light red solution was kept for evaporating. Some red single crystals were obtained about 19 days later.

### Refinement

The structure was solved by direct methods and successive Fourier difference synthesis. The H atoms bonded to C atoms were positioned geometrically and refined using a riding model [aromatic C—H = 0.93 Å (*U*_iso_(H) = 1.2*U*_eq_(C))]. The H atoms bonded to O atoms were located in difference Fourier maps and refined with O—H distance restraints of 0.85 (2) and *U*_iso_(H) = 1.5*U*_eq_(O).

### Crystal data

**Table:**

C_10_H_7_NO_2_Se	*F*(000) = 496
*M**_r_* = 252.13	*D*_x_ = 1.732 Mg m^−^^3^
Monoclinic, *P*2_1_/*c*	Mo *K*α radiation, λ = 0.71073 Å
Hall symbol: -P 2ybc	Cell parameters from 4000 reflections
*a* = 8.0817 (3) Å	θ = 2.6–25.0°
*b* = 11.5661 (4) Å	µ = 3.85 mm^−^^1^
*c* = 11.6295 (4) Å	*T* = 296 K
β = 117.168 (2)°	Block, red
*V* = 967.12 (6) Å^3^	0.23 × 0.22 × 0.19 mm
*Z* = 4	

### Data collection

**Table:**

Bruker APEXII area-detector diffractometer	1705 independent reflections
Radiation source: fine-focus sealed tube	1487 reflections with *I* > 2σ(*I*)
graphite	*R*_int_ = 0.022
Detector resolution: none pixels mm^-1^	θ_max_ = 25.0°, θ_min_ = 2.6°
φ and ω scans	*h* = −9→9
Absorption correction: multi-scan (*SADABS*; Sheldrick, 1996)	*k* = −13→13
*T*_min_ = 0.437, *T*_max_ = 0.479	*l* = −13→13
7502 measured reflections	

### Refinement

**Table:**

Refinement on *F*^2^	Primary atom site location: structure-invariant direct methods
Least-squares matrix: full	Secondary atom site location: difference Fourier map
*R*[*F*^2^ > 2σ(*F*^2^)] = 0.024	Hydrogen site location: inferred from neighbouring sites
*wR*(*F*^2^) = 0.063	H-atom parameters constrained
*S* = 1.05	*w* = 1/[σ^2^(*F*_o_^2^) + (0.0341*P*)^2^ + 0.3951*P*] where *P* = (*F*_o_^2^ + 2*F*_c_^2^)/3
1705 reflections	(Δ/σ)_max_ = 0.001
127 parameters	Δρ_max_ = 0.43 e Å^−^^3^
0 restraints	Δρ_min_ = −0.18 e Å^−^^3^

### Special details

**Table:**

Geometry. All e.s.d.'s (except the e.s.d. in the dihedral angle between two l.s. planes) are estimated using the full covariance matrix. The cell e.s.d.'s are taken into account individually in the estimation of e.s.d.'s in distances, angles and torsion angles; correlations between e.s.d.'s in cell parameters are only used when they are defined by crystal symmetry. An approximate (isotropic) treatment of cell e.s.d.'s is used for estimating e.s.d.'s involving l.s. planes.
Refinement. Refinement of *F*^2^ against ALL reflections. The weighted *R*-factor *wR* and goodness of fit *S* are based on *F*^2^, conventional *R*-factors *R* are based on *F*, with *F* set to zero for negative *F*^2^. The threshold expression of *F*^2^ > σ(*F*^2^) is used only for calculating *R*-factors(gt) *etc*. and is not relevant to the choice of reflections for refinement. *R*-factors based on *F*^2^ are statistically about twice as large as those based on *F*, and *R*- factors based on ALL data will be even larger.

### Fractional atomic coordinates and isotropic or equivalent isotropic displacement parameters (Å^2^)

**Table:**

	*x*	*y*	*z*	*U*_iso_*/*U*_eq_	
Se1	0.26583 (4)	−0.04306 (2)	0.60598 (2)	0.04992 (12)	
O1	0.4357 (3)	−0.41679 (16)	0.5986 (2)	0.0685 (6)	
H7	0.4714	−0.4799	0.5865	0.103*	
O2	0.4478 (3)	−0.37486 (15)	0.41673 (18)	0.0661 (6)	
N1	0.3215 (3)	−0.15135 (15)	0.42195 (18)	0.0382 (4)	
C1	0.1846 (4)	0.0295 (2)	0.2299 (3)	0.0543 (7)	
H1	0.2010	−0.0436	0.2033	0.065*	
C2	0.1285 (4)	0.1208 (2)	0.1426 (3)	0.0655 (8)	
H2	0.1085	0.1088	0.0581	0.079*	
C3	0.1028 (4)	0.2287 (2)	0.1809 (3)	0.0595 (7)	
H3	0.0630	0.2894	0.1220	0.071*	
C4	0.1357 (4)	0.2467 (2)	0.3054 (3)	0.0635 (8)	
H4	0.1201	0.3201	0.3315	0.076*	
C5	0.1921 (4)	0.1566 (2)	0.3930 (3)	0.0538 (6)	
H5	0.2141	0.1696	0.4778	0.065*	
C6	0.2159 (3)	0.04648 (18)	0.3548 (2)	0.0388 (5)	
C7	0.2713 (3)	−0.05191 (17)	0.4454 (2)	0.0364 (5)	
C8	0.3408 (3)	−0.1944 (2)	0.6245 (2)	0.0438 (5)	
H8	0.3632	−0.2404	0.6957	0.053*	
C9	0.3587 (3)	−0.22941 (18)	0.5206 (2)	0.0386 (5)	
C10	0.4173 (4)	−0.3472 (2)	0.5075 (2)	0.0455 (6)	

### Atomic displacement parameters (Å^2^)

**Table:**

	*U*^11^	*U*^22^	*U*^33^	*U*^12^	*U*^13^	*U*^23^
Se1	0.0692 (2)	0.04354 (17)	0.04006 (17)	0.01159 (11)	0.02764 (14)	0.00007 (10)
O1	0.1212 (18)	0.0434 (9)	0.0619 (13)	0.0252 (11)	0.0600 (13)	0.0203 (9)
O2	0.1187 (17)	0.0448 (10)	0.0514 (12)	0.0251 (10)	0.0533 (12)	0.0128 (8)
N1	0.0453 (11)	0.0337 (10)	0.0362 (10)	0.0043 (8)	0.0191 (8)	0.0046 (8)
C1	0.0716 (18)	0.0431 (14)	0.0488 (16)	0.0075 (12)	0.0281 (14)	0.0073 (11)
C2	0.084 (2)	0.0629 (18)	0.0483 (17)	0.0095 (15)	0.0292 (15)	0.0149 (14)
C3	0.0576 (16)	0.0517 (15)	0.068 (2)	0.0135 (12)	0.0281 (14)	0.0258 (14)
C4	0.0721 (19)	0.0394 (14)	0.084 (2)	0.0153 (13)	0.0404 (17)	0.0128 (13)
C5	0.0674 (17)	0.0421 (14)	0.0571 (17)	0.0113 (12)	0.0331 (14)	0.0054 (11)
C6	0.0363 (12)	0.0367 (12)	0.0440 (13)	0.0025 (9)	0.0187 (10)	0.0044 (9)
C7	0.0371 (11)	0.0357 (11)	0.0354 (12)	0.0012 (9)	0.0157 (9)	0.0013 (9)
C8	0.0543 (14)	0.0412 (12)	0.0357 (13)	0.0048 (11)	0.0204 (11)	0.0041 (10)
C9	0.0438 (13)	0.0357 (11)	0.0360 (12)	0.0027 (9)	0.0179 (10)	0.0031 (9)
C10	0.0620 (15)	0.0376 (12)	0.0404 (13)	0.0061 (11)	0.0264 (12)	0.0063 (10)

### Geometric parameters (Å, °)

**Table:**

Se1—C8	1.832 (2)	C2—H2	0.9300
Se1—C7	1.891 (2)	C3—C4	1.363 (4)
O1—C10	1.284 (3)	C3—H3	0.9300
O1—H7	0.8201	C4—C5	1.382 (4)
O2—C10	1.230 (3)	C4—H4	0.9300
N1—C7	1.289 (3)	C5—C6	1.391 (3)
N1—C9	1.381 (3)	C5—H5	0.9300
C1—C6	1.370 (4)	C6—C7	1.474 (3)
C1—C2	1.390 (4)	C8—C9	1.344 (3)
C1—H1	0.9300	C8—H8	0.9300
C2—C3	1.372 (4)	C9—C10	1.473 (3)
			
C8—Se1—C7	84.79 (10)	C6—C5—H5	119.9
C10—O1—H7	109.5	C1—C6—C5	119.0 (2)
C7—N1—C9	112.19 (19)	C1—C6—C7	119.7 (2)
C6—C1—C2	120.4 (2)	C5—C6—C7	121.3 (2)
C6—C1—H1	119.8	N1—C7—C6	124.0 (2)
C2—C1—H1	119.8	N1—C7—Se1	114.10 (16)
C3—C2—C1	120.1 (3)	C6—C7—Se1	121.82 (15)
C3—C2—H2	120.0	C9—C8—Se1	110.35 (17)
C1—C2—H2	120.0	C9—C8—H8	124.8
C4—C3—C2	119.9 (2)	Se1—C8—H8	124.8
C4—C3—H3	120.0	C8—C9—N1	118.6 (2)
C2—C3—H3	120.0	C8—C9—C10	123.0 (2)
C3—C4—C5	120.5 (3)	N1—C9—C10	118.48 (19)
C3—C4—H4	119.8	O2—C10—O1	123.5 (2)
C5—C4—H4	119.8	O2—C10—C9	121.9 (2)
C4—C5—C6	120.1 (3)	O1—C10—C9	114.6 (2)
C4—C5—H5	119.9		
			
C6—C1—C2—C3	−0.5 (5)	C5—C6—C7—Se1	12.7 (3)
C1—C2—C3—C4	1.3 (5)	C8—Se1—C7—N1	−0.22 (18)
C2—C3—C4—C5	−1.0 (4)	C8—Se1—C7—C6	177.7 (2)
C3—C4—C5—C6	0.1 (4)	C7—Se1—C8—C9	−0.05 (18)
C2—C1—C6—C5	−0.5 (4)	Se1—C8—C9—N1	0.3 (3)
C2—C1—C6—C7	178.7 (3)	Se1—C8—C9—C10	179.96 (19)
C4—C5—C6—C1	0.7 (4)	C7—N1—C9—C8	−0.5 (3)
C4—C5—C6—C7	−178.4 (2)	C7—N1—C9—C10	179.8 (2)
C9—N1—C7—C6	−177.5 (2)	C8—C9—C10—O2	−173.9 (3)
C9—N1—C7—Se1	0.4 (2)	N1—C9—C10—O2	5.8 (4)
C1—C6—C7—N1	11.4 (3)	C8—C9—C10—O1	5.3 (4)
C5—C6—C7—N1	−169.5 (2)	N1—C9—C10—O1	−175.1 (2)
C1—C6—C7—Se1	−166.39 (19)		

### Hydrogen-bond geometry (Å, °)

**Table:**

*D*—H···*A*	*D*—H	H···*A*	*D*···*A*	*D*—H···*A*
O1—H7···O2^i^	0.82	1.81	2.623 (2)	171

Symmetry codes: (i) −*x*+1, −*y*−1, −*z*+1.
